# *Lactobacillus johnsonii* ameliorates intestinal, extra-intestinal and systemic pro-inflammatory immune responses following murine *Campylobacter jejuni* infection

**DOI:** 10.1038/s41598-017-02436-2

**Published:** 2017-05-18

**Authors:** Stefan Bereswill, Ira Ekmekciu, Ulrike Escher, Ulrike Fiebiger, Kerstin Stingl, Markus M. Heimesaat

**Affiliations:** 1 Department of Microbiology and Hygiene, Charité - University Medicine, Berlin, Germany; 2 Federal Institute for Risk Assessment (BfR), Department of Biological Safety, National Reference Laboratory for Campylobacter, Berlin, Germany

## Abstract

*Campylobacter jejuni* infections are progressively increasing worldwide. Probiotic treatment might open novel therapeutic or even prophylactic approaches to combat campylobacteriosis. In the present study secondary abiotic mice were generated by broad-spectrum antibiotic treatment and perorally reassociated with a commensal murine *Lactobacillus johnsonii* strain either 14 days before (i.e. prophylactic regimen) or 7 days after (i.e. therapeutic regimen) peroral *C*. *jejuni* strain 81–176 infection. Following peroral reassociation both *C*. *jejuni* and *L*. *johnsonii* were able to stably colonize the murine intestinal tract. Neither therapeutic nor prophylactic *L*. *johnsonii* application, however, could decrease intestinal *C*. *jejuni* burdens. Notably, *C*. *jejuni* induced colonic apoptosis could be ameliorated by prophylactic *L*. *johnsonii* treatment, whereas co-administration of *L*. *johnsonii* impacted adaptive (i.e. T and B lymphocytes, regulatory T cells), but not innate (i.e. macrophages and monocytes) immune cell responses in the intestinal tract. Strikingly, *C*. *jejuni* induced intestinal, extra-intestinal and systemic secretion of pro-inflammatory mediators (such as IL-6, MCP-1, TNF and nitric oxide) could be alleviated by peroral *L*. *johnsonii* challenge. In conclusion, immunomodulatory probiotic species might offer valuable strategies for prophylaxis and/or treatment of *C*. *jejuni* induced intestinal, extra-intestinal as well as systemic pro-inflammatory immune responses *in vivo*.

## Introduction

Human *Campylobacter jejuni* infections are progressively rising worldwide^1, 2^. Whereas *C*. *jejuni* act as commensal bacteria within the intestinal tract of wild and domestic animals, humans acquire *C*. *jejuni* usually by consumption of contaminated products derived from livestock animals or contaminated surface water via the peroral route and present with clinical symptoms of varying degree^3–6^. Whereas some patients suffer from rather mild malaise, others present with gastroenteritis ranging from watery diarrhea to severe ulcerative colitis with inflammatory, bloody diarrhea^7^. Whereas in the vast majority of cases disease resolves spontaneously, post-infectious sequelae including peripheral neuropathies such as Guillain-Barré and Miller-Fisher syndromes and reactive arthritis might develop with a latency of weeks to months^8–10^. Due to the scarcity of appropriate *in vivo* models, our understanding of the molecular mechanisms underlying *Campylobacter*-host interactions has been hampered for a long time^6, 11^. In general, mice are highly convenient for studies of bacterial pathogenicity and pathogen-host interactions. Conventionally colonized mice, however, are protected from *C*. *jejuni* infection due to their host specific microbiota composition mediating physiological colonization resistance^6, 12^. Our group showed previously that *C*. *jejuni* infection could be facilitated by modification of the murine intestinal microbiota^6, 12, 13^. Physiological colonization resistance could be overcome upon virtual eradication of the intestinal microbiota by broad-spectrum antibiotic treatment. These secondary abiotic mice could be stably infected with the pathogen and exhibited key features of human campylobacteriosis including apoptosis and pro-inflammatory immune responses in the large intestines^12^. Hence, secondary abiotic mice are well-suited to unravel the triangle relationship between intestinal pathogens, commensals and the host immune system *in vivo* ^14^. These findings suggest that microorganisms in the gut microbiota of our laboratory mice might contribute to colonization resistance against *C*. *jejuni*, thus providing health benefits to the host similar to probiotics^15^.

It is well-established that modulation of the intestinal microbiota composition by probiotic microbes is a promising strategy to prevent pathogenic colonization and subsequent infection in susceptible hosts^15, 16^. The reduction of the *C*. *jejuni* loads in poultry and other livestock animals is a highly preventive measure to prevent campylobacteriosis in humans. The molecular mechanisms inhibiting *C*. *jejuni* colonization are in the actual focus of research. In this context the extraordinary colonization resistance displayed by our conventional mice against *C*. *jejuni* raises intensive scientific interest. The murine intestinal microbiota is dominated by Gram-positive lactobacilli belonging to the *Lacobacillaceae* of the *Lactobacillales* within the *Bacilli* class of the *Firmicutes* ^16, 17^. The genus is the most diverse amongst the lactic acid bacteria, and a variety of probiotic lactobacilli including *L*. *acidophilus*/*L*. *johnsonii*, *L*. *casei*, *L*. *rhamnosus*, *L*. *gasseri*, and closely related *Bifidobacterium lactis* have been shown to inhibit virulence of enteropathogenic bacteria including *Campylobacter*, *Salmonella*, *Shigella*, enterotoxigenic *E*. *coli* or *Vibrio cholerae* to intestinal cells^18–22^. In particular for *C*. *jeuni* it has been conclusively demonstrated that *L*. *gasseri* SBT2055 inhibits invasion of epithelial cells by co-aggregation *in vitro* and counteracts pathogenic colonization in chicken^23, 24^. Furthermore, production of organic acids (mainly lactic acid) by probiotic lactobacilli exerts killing of *C*. *jejuni in vitro* and is efficiently reducing *C*. *jejuni* concentrations *in vivo* as shown in poultry^25^. Whereas *Lactobacillus* spp. isolated from probiotic formulations were highly effective in mediating colonization resistance against *C*. *jejuni* in mice^26^, bacteriocin producing *L*. *salivarius* reduced *C*. *jejuni* loads in the chicken intestinal tract^27^.

Given that distinct species in the dense *Lactobacillus* population in mice might be involved in mediating colonization resistance against *C*. *jejuni*, we isolated a commensal *Lactobacillus johnsonii* strain from murine fecal samples (see methods) and confirmed its antimicrobial activity against *C*. *jejuni* by using co-cultivation assays *in vitro* (Bachelor thesis Ulrike Escher, 2014 Beuth Hochschule, Berlin, Germany). The inhibition of *C*. *jejuni* growth by *L*. *johnsonii* was mediated via production of organic acids, as demonstrated by the loss of the antimicrobial activity against *C*. *jejuni* by neutralization of the growth media with alkalizing agents. Notably *L*. *johnsonii* (formerly termed *L*. *acidophilus*) constitutes a common commensal of the murine intestinal microbiota^28^. Given its scientifically confirmed probiotic potential, several *L*. *johnsonii* strains are used worldwide in probiotic products^29^. Most importantly, *L*. *johnsonii* inhibits the growth of *C*. *jejuni in vitro* as already known for decades^30^.

In the present study we investigated the potential probiotic/beneficial effects of *L*. *johnsonii* in murine campylobacteriosis by prophylactic and therapeutic treatment of *C*. *jejuni* infected secondary abiotic mice which were generated by antibiotic treatment. Results obtained by analysis of the resulting secondary abiotic animals revealed that prophylactic or therapeutic treatment of mice with *L*. *johnsonii* did not lower intestinal *C*. *jejuni* colonization, but significantly suppressed intestinal and systemic pro-inflammatory and enhanced anti-inflammatory immune responses.

## Results

### Intestinal colonization capacities of *L*. *johnsonii* and/or *C*. *jejuni* strain 81–176 in perorally reassociated secondary abiotic mice

**Figure 1:**
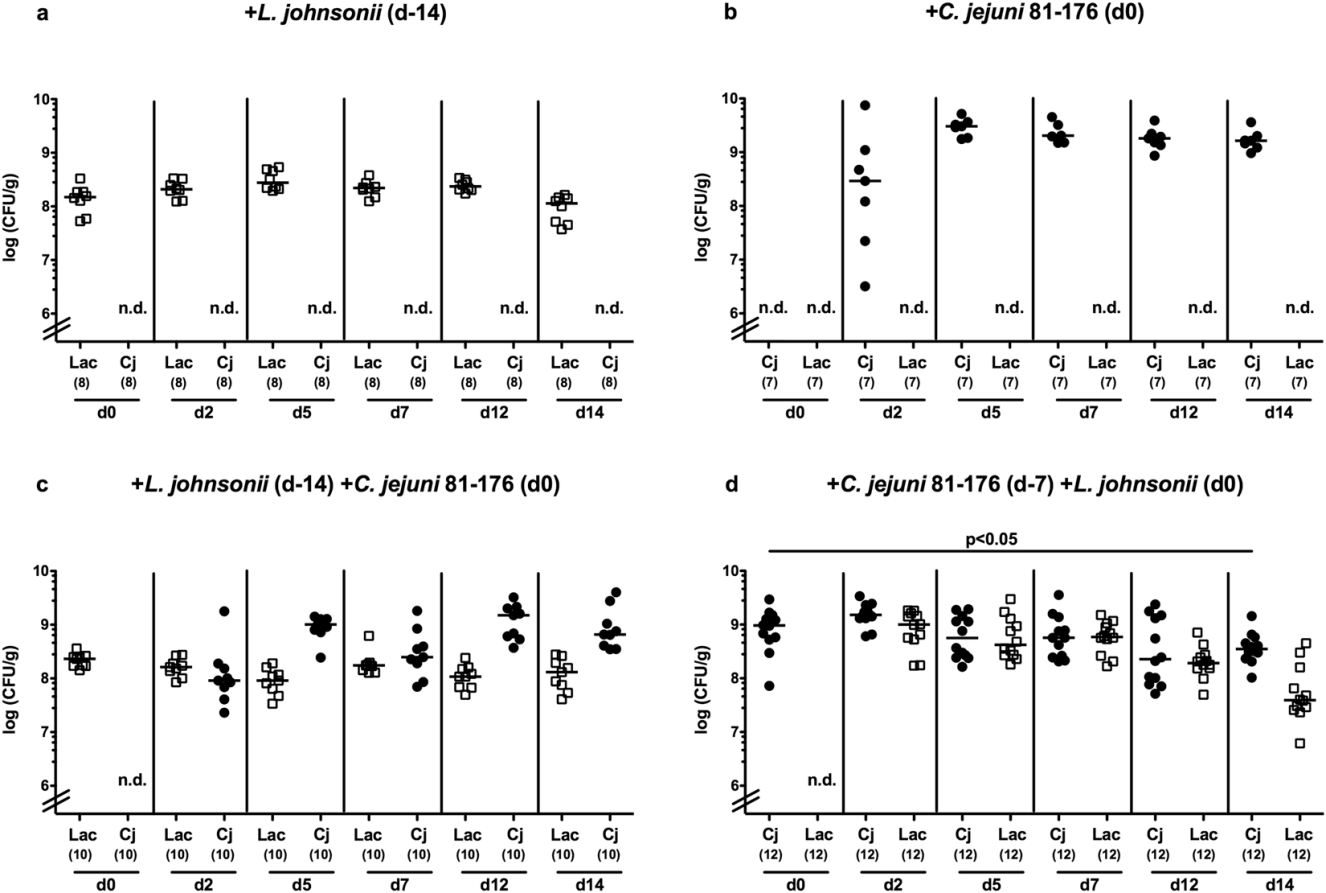
Kinetics of intestinal *L*. *johnsonii* and/or *C*. *jejuni* strain 81–176 loads in perorally reassociated secondary abiotic mice. Secondary abiotic mice were generated by broad-spectrum antibiotic treatment and perorally reassociated with (**a**) *L*. *johnsonii* (Lac; white squares) on day (**d**) −14, (**b**) *C*. *jejuni* strain 81–176 (Cj; black circles) on d0, (**c**) *L*. *johnsonii* on day −14 and *C*. *jejuni* strain 81–176 on d0, or (**d**) *C*. *jejuni* strain 81–176 (d-7) and *L*. *johnsonii* on d0 as described in methods. Bacterial colonization densities were assessed in fecal samples (CFU/g, colony forming units per gram) over time upon reassociation as indicated by culture. Medians (black bars) and levels of significance (p-value) determined by Mann-Whitney U test are indicated. Numbers of analyzed mice are given in parentheses. Data were pooled from three independent experiments. N.d.: not determined.

In the present study we investigated the potential of a murine commensal intestinal *L*. *johnsonii* strain to reduce intestinal pathogenic burdens and to alleviate pro-inflammatory immune responses upon *C*. *jejuni* infection *in vivo*. In order to overcome physiological colonization resistance exerted by the conventional murine microbiota and to assure subsequent stable intestinal bacterial colonization^12^, we subjected conventionally raised and housed mice to broad-spectrum antibiotic treatment for eight weeks. Consequently, the murine intestinal microbiota was virtually depleted. These generated secondary abiotic mice were then perorally challenged with 10^8^ colony forming units (CFU) of a *L*. *johnsonii* strain, that had initially been isolated from the commensal intestinal microbiota of a healthy mouse, by gavage either 14 days before (i.e. prophylactic regimen) or 7 days after (i.e. therapeutic regimen) peroral *C*. *jejuni* strain 81–176 infection (pathogenic loads of 10^8^ CFU). At day 28 and day 21 following initial *L*. *johnsonii* and *C*. *jejuni* reassociation, respectively, mice from bacterial *in vivo* competition experiments were compared to mono-associated and naive mice. Following peroral challenge, either bacterial strain was able to stably colonize the murine intestinal tract with high median loads of approximately 10^8^ to 10^9^ CFU per gram feces (Fig. 1). Within 14 days following *L*. *johnsonii* coassociation of *C*. *jejuni* infected mice, fecal pathogenic burdens could be lowered by less than 0.5 order of magnitude (p < 0.05; Fig. 1d). Overall, both *C*. *jejuni* and *L*. *johnsonii* stably colonized the intestinal tract of secondary abiotic mice at comparable loads. Neither therapeutic nor prophylactic *L*. *johnsonii* application, however, was able to decrease intestinal pathogenic burdens in a biologically relevant fashion (Fig. 1c,d).

### Large intestinal apoptosis following reassociation of secondary abiotic mice with *C*. *jejuni* and/or *L*. *johnsonii*

**Figure 2:**
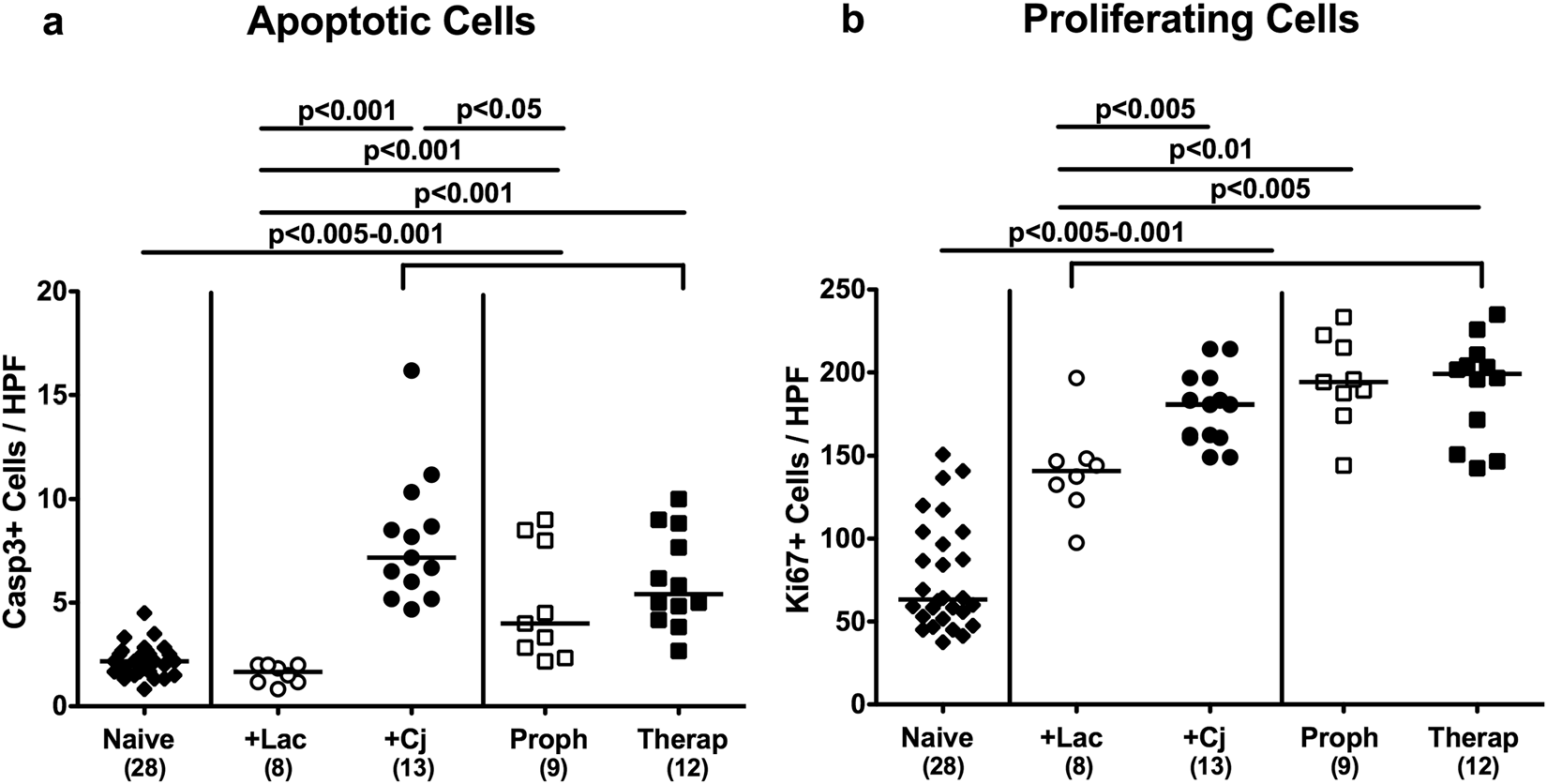
Apoptotic and proliferating cells in the colonic epithelium of *C*. *jejuni* strain 81–176 and/or *L*. *johnsonii* associated secondary abiotic mice. Secondary abiotic mice were perorally infected with *C*. *jejuni* strain 81–176 (Cj) and associated with *L*. *johnsonii* (Lac) either 14 days before (prophylactic regimen, Proph; white squares) or 7 days thereafter (therapeutic regimen, Therap; black squares) and compared to mono-associated mice (+Lac, white circles; +Cj, black circles). The average number of colonic (**a**) apoptotic cells (positive for caspase-3, Casp3) and (**b**) proliferating cells (positive for Ki67) from six high power fields (HPF, 400x magnification) per animal was determined microscopically in immunohistochemically stained colonic paraffin sections at days 21 or 28 following initial *C*. *jejuni* or *L*. *johnsonii* infection, respectively. Naive mice (black diamonds) served as uninfected controls. Medians (black bars), levels of significance (p-values) determined by one-way ANOVA test followed by Tukey post-correction test for multiple comparisons and numbers of analyzed animals (in parentheses) are indicated. Data were pooled from three independent experiments.

Since reassociation of secondary abiotic mice with *C*. *jejuni* and/or *L*. *johnsonii* did not affect mice macroscopically (i.e. clinically; not shown), we next investigated potential microscopic sequelae of respective bacterial challenges. Apoptosis is a well-established marker for histopathological grading of intestinal inflammation and a key feature of campylobacteriosis^12^. We therefore quantitatively assessed caspase3 positive cell numbers in colonic epithelia applying *in situ* immunohistochemistry. *C*. *jejun*i infected mice exhibited increased numbers of apoptotic cells in their large intestines as compared to naive and *L*. *johnsonii* mono-associated mice (p < 0.005–0.001; Fig. 2a; Supplemental Fig. S1). Prophylactic administration of *L*. *johnsonii*, however, was associated with approximately 50% lower apoptotic colonic epithelial cell numbers as compared to *C*. *jejuni* mono-associated mice (p < 0.05; Fig. 2a; Supplemental Fig. S1), whereas a trend towards less apoptosis could also be observed in the *L*. *johnsonii* treatment group (n.s.; Fig. 2a; Supplemental Fig. S1). Notably, *L*. *johnsonii* application alone did not induce colonic apoptosis (n.s. vs naive controls; Fig. 2a; Supplemental Fig. S1). Reassociation of gnotobiotic mice with *C*. *jejuni* and/or *L*. *johnsonii* was further accompanied by increases in colonic numbers of Ki67 positive cells indicative for cell proliferation and regeneration counteracting potential *C*. *jejuni* induced epithelial cell damage (p < 0.005–0.001; Fig. 2b; Supplemental Fig. S1). Hence, *C*. *jejuni* induced colonic apoptosis could be ameliorated by pre-treatment with *L*. *johnsonii*.

### Large intestinal innate and adaptive immune cell responses upon reassociation of secondary abiotic mice with *C*. *jejuni* and/or *L*. *johnsonii*

**Figure 3:**
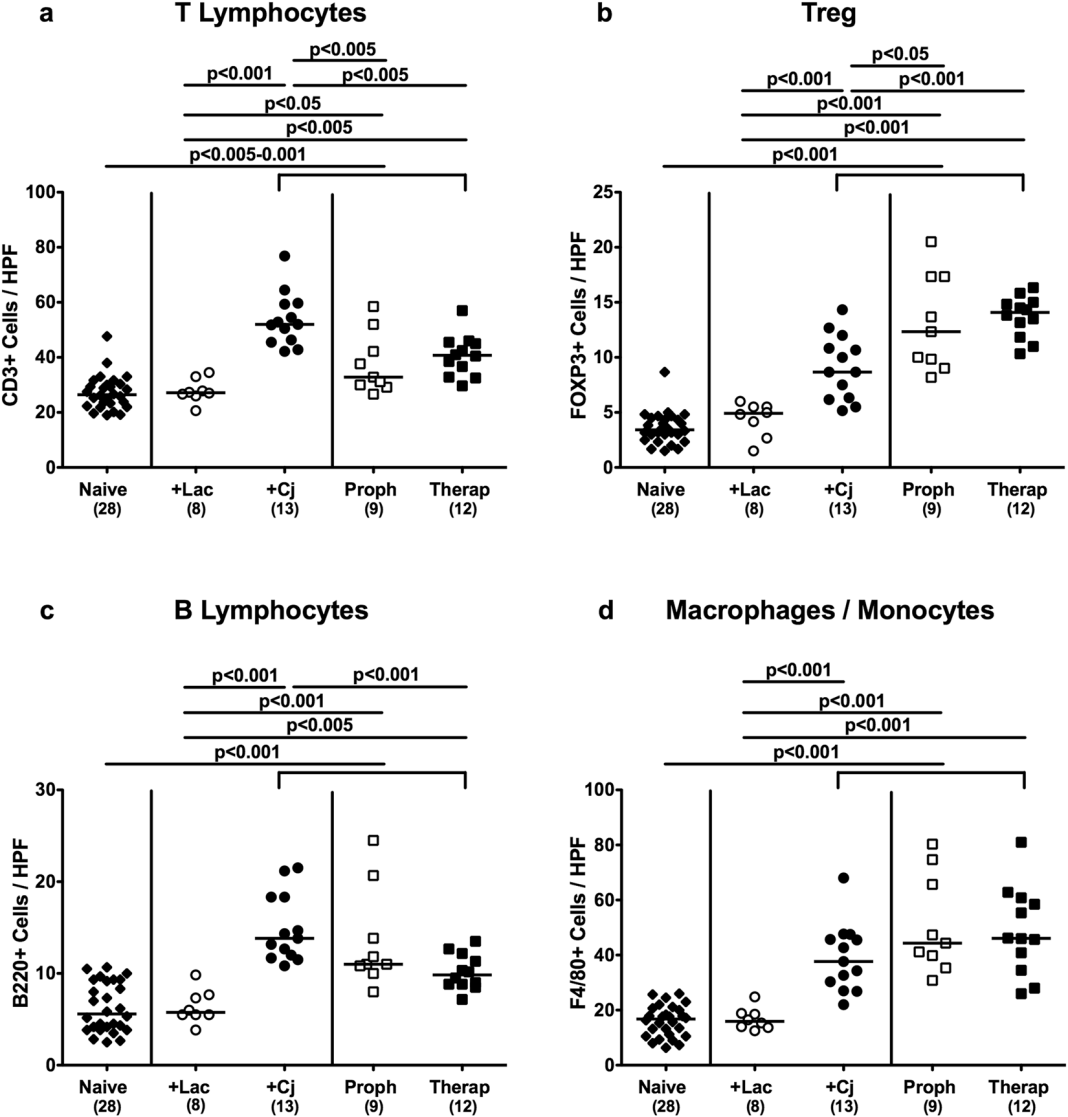
Colonic immune cell responses in *C. jejuni* strain 81–176 and/or *L. johnsonii* reassociated secondary abiotic mice. Secondary abiotic mice were perorally infected with *C*. *jejuni* strain 81–176 (Cj) and associated with *L*. *johnsonii* (Lac) either 14 days before (prophylactic regimen, Proph; white squares) or 7 days thereafter (therapeutic regimen, Therap; black squares) and compared to mono-associated mice (+Lac, white circles; +Cj, black circles). The average number of colonic (**a**) T lymphocytes (positive for CD3), (**b**) regulatory T cells (Treg; positive for FOXP3), (**c**) B lymphocytes (positive for B220), and (**d**) macrophages and monocytes (positive for F4/80) from six high power fields (HPF, 400x magnification) per animal was determined microscopically in immunohistochemically stained colonic paraffin sections at days 21 or 28 following initial *C*. *jejuni* or *L*. *johnsonii* infection, respectively. Naive mice (black diamonds) served as uninfected controls. Medians (black bars), levels of significance (p-values) determined by one-way ANOVA test followed by Tukey post-correction test for multiple comparisons and numbers of analyzed animals (in parentheses) are indicated. Data were pooled from three independent experiments.

Recruitment of pro-inflammatory immune cells to the site of infection is a characteristic feature of intestinal inflammation including campylobacteriosis^12^. We therefore quantitatively determined distinct innate as well as adaptive immune cell subsets in large intestinal *ex vivo* biopsies by *in situ* immunohistochemistry. Peroral *C*. *jejuni* infection, but not *L*. *johnsonii* application alone resulted in increased colonic numbers of T and B lymphocytes, regulatory T cells (Treg) as well as macrophages and monocytes (p < 0.001; Fig. 3; Supplemental Fig. S1). Colonic T lymphocyte numbers, however, decreased (p < 0.005), whereas conversely, Treg counts increased (p < 0.05–0.001) in bacterial competition experiments as compared to mice subjected to *C*. *jejuni* alone, irrespective whether *L*. *johnsonii* was applied prophylactically or therapeutically (Fig. 3a,b; Supplemental Fig. S1). Furthermore therapeutic, but not prophylactic *L*. *johnsonii* treatment of *C*. *jejuni* infected mice resulted in decreased colonic B lymphocyte numbers (p < 0.001; Fig. 3c; Supplemental Fig. S1). Notably, large intestinal counts of macrophages and monocytes were not affected by *L*. *johnsonii* co-association of *C*. *jejuni* infected animals (n.s.; Fig. 3d; Supplemental Fig. S1).

### Intestinal cytokine responses upon reassociation of secondary abiotic mice with *C*. *jejuni* and/or *L*. *johnsonii*

**Figure 4:**
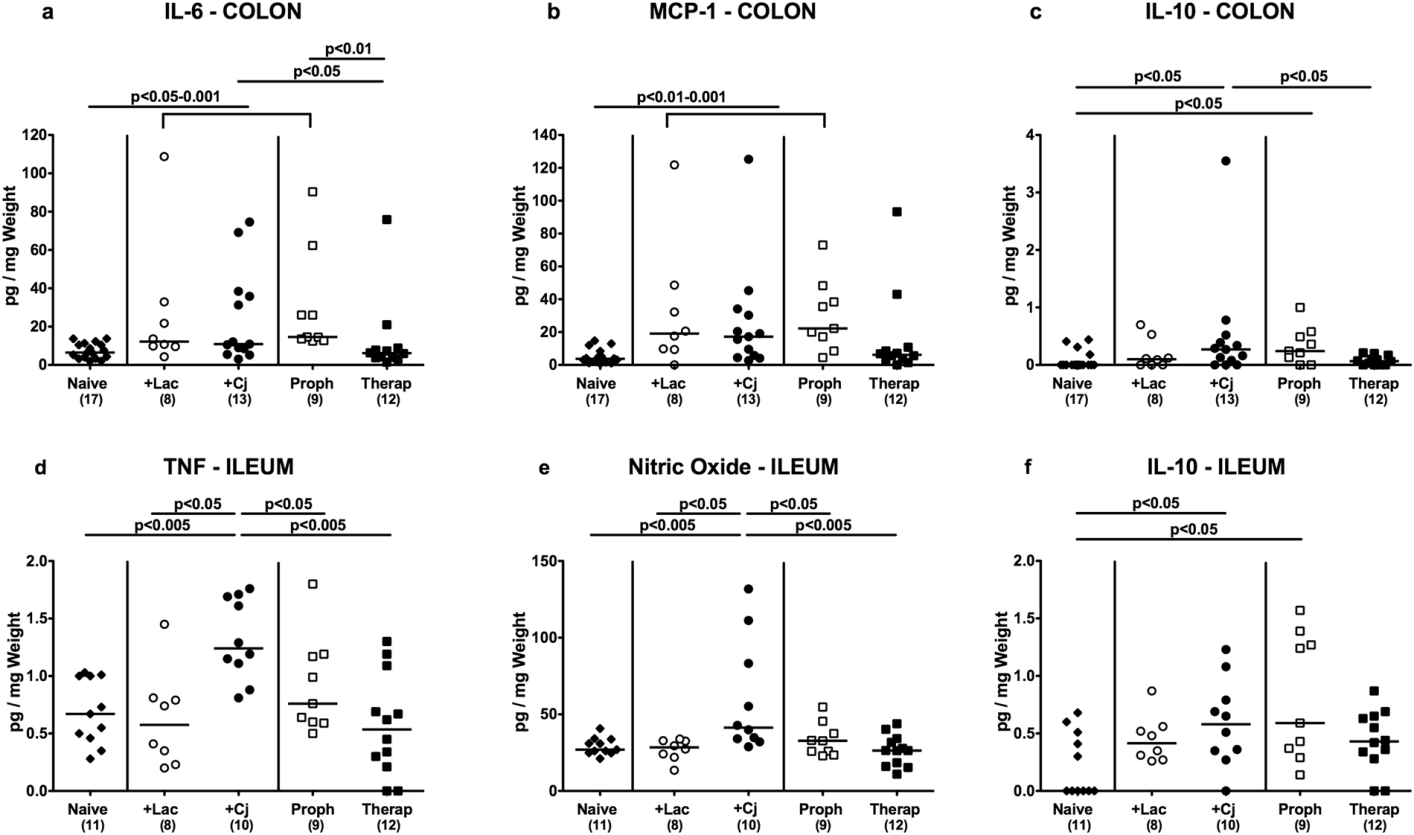
Intesinal secretion of pro- and anti-inflammatory mediators in *C*. *jejuni* strain 81–176 and/or *L*. *johnsonii* reassociated secondary abiotic mice. Secondary abiotic mice were perorally infected with *C*. *jejuni* strain 81–176 (Cj) and associated with *L*. *johnsonii* (Lac) either 14 days before (prophylactic regimen, Proph; white squares) or 7 days thereafter (therapeutic regimen, Therap; black squares) and compared to mono-associated mice (+Lac, white circles; +Cj, black circles). Colonic (**a**) IL-6, (**b**) MCP-1 and (**c**) IL-10 concentrations as well as ileal (**d**) TNF, (**e**) nitric oxide and (**f**) IL-10 secretion were determined in *ex vivo* biopsies derived at days 21 or 28 following initial *C*. *jejuni* or *L*. *johnsonii* infection, respectively. Naive (N) mice (black diamonds) served as uninfected controls. Medians (black bars), levels of significance (p-value) determined by one-way ANOVA test followed by Tukey post-correction test for multiple comparisons and numbers of analyzed animals (in parentheses) are indicated. Data were pooled from three independent experiments.

We further measured pro- and anti-inflammatory cytokine secretion in distinct intestinal compartments generated in bacterial *in vivo* competition experiments. In colonic *ex vivo* biopsies, IL-6 and MCP-1 concentrations increased following mono-association of secondary abiotic mice with *L*. *johnsonii* or *C*. *jejuni*, which was also true for prophylactically with *L*. *johnsonii* treated *C*. *jejuni* infected animals (p < 0.05–0.001; Fig. 4a,b). Notably, colonic secretion of respective pro-inflammatory cytokines was similar in naive and *C*. *jejuni* associated mice that were therapeutically treated with *L*. *johnsonii* (n.s.; Fig. 4a,b). Furthermore, colonic levels of the anti-inflammatory cytokine IL-10 increased upon *C*. *jejuni* infection, except for therapeutically with *L*. *johnsonii* treated animals (p < 0.05; Fig. 4c).

Even though *C*. *jejuni* has been known to primarily affect the large intestines^11^, we expanded our cytokine measurements to the small intestinal tract. Remarkably, *C*. *jejuni* induced increases in ileal TNF and nitric oxide (NO) secretion could be dampened to naive levels by both prophylactic and therapeutic *L*. *johnsonii* administration (p < 0.05 and p < 0.005, respectively; Fig. 4d,e). Like in the colon, IL-10 concentrations increased in the distal small intestinal tract upon *C*. *jejuni* mono-association as well as in *C*. *jejuni* infected mice that were prophylactically treated with *L*. *johnsonii* (p < 0.01 and p < 0.05, respectively; Fig. 4f).

**Figure 5:**
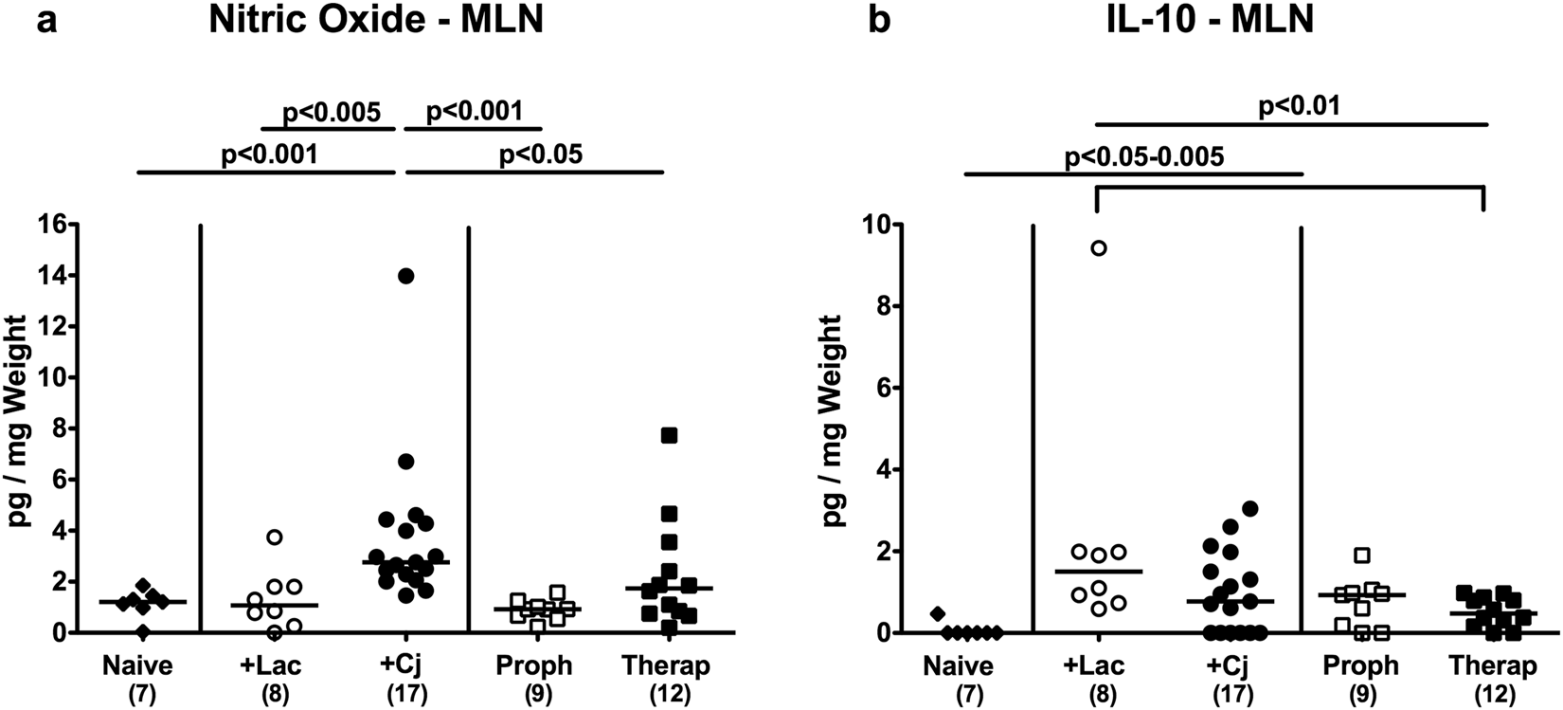
Secretion of pro- and anti-inflammatory mediators in mesenteric lymph nodes of *C*. *jejuni* strain 81–176 and/or *L*. *johnsonii* reassociated secondary abiotic mice. Secondary abiotic mice were perorally infected with *C*. *jejuni* strain 81–176 (Cj) and associated with *L*. *johnsonii* (Lac) either 14 days before (prophylactic regimen, Proph; white squares) or 7 days thereafter (therapeutic regimen, Therap; black squares) and compared to mono-associated mice (+Lac, white circles; +Cj, black circles). (**a**) Nitric oxide and (**b**) IL-10 concentrations were determined in *ex vivo* biopsies derived from mesenteric lymph nodes (MLN) at days 21 or 28 following initial *C*. *jejuni* or *L*. *johnsonii* infection, respectively. Naive (N) mice (black diamonds) served as uninfected controls. Medians (black bars), level of significance (p-value) determined by one-way ANOVA test followed by Tukey post-correction test for multiple comparisons and numbers of analyzed animals (in parentheses) are indicated. Data were pooled from three independent experiments.

Furthermore, NO levels were increased in mesenteric lymph nodes (MLN) of *C*. *jejuni* infected mice (p < 0.005), but could be reduced to basal (naive) levels by *L*. *johnsonii* co-administration (p < 0.05–0.001; Fig. 5a). Upon bacterial reassociation of either regimen, elevated IL-10 concentrations could be measured in MLN of secondary abiotic mice (p < 0.05–0.005; Fig. 5b).

Hence, *C*. *jejuni* induced increases in intestinal pro-inflammatory cytokine secretion could be alleviated by peroral *L*. *johnsonii* challenge. Notably, respective beneficial immunomodulatory effects were not restricted to the large intestinal tract.

### Extra-intestinal cytokine responses upon reassociation of secondary abiotic mice with *C*. *jejuni* and/or *L*. *johnsonii*

**Figure 6:**
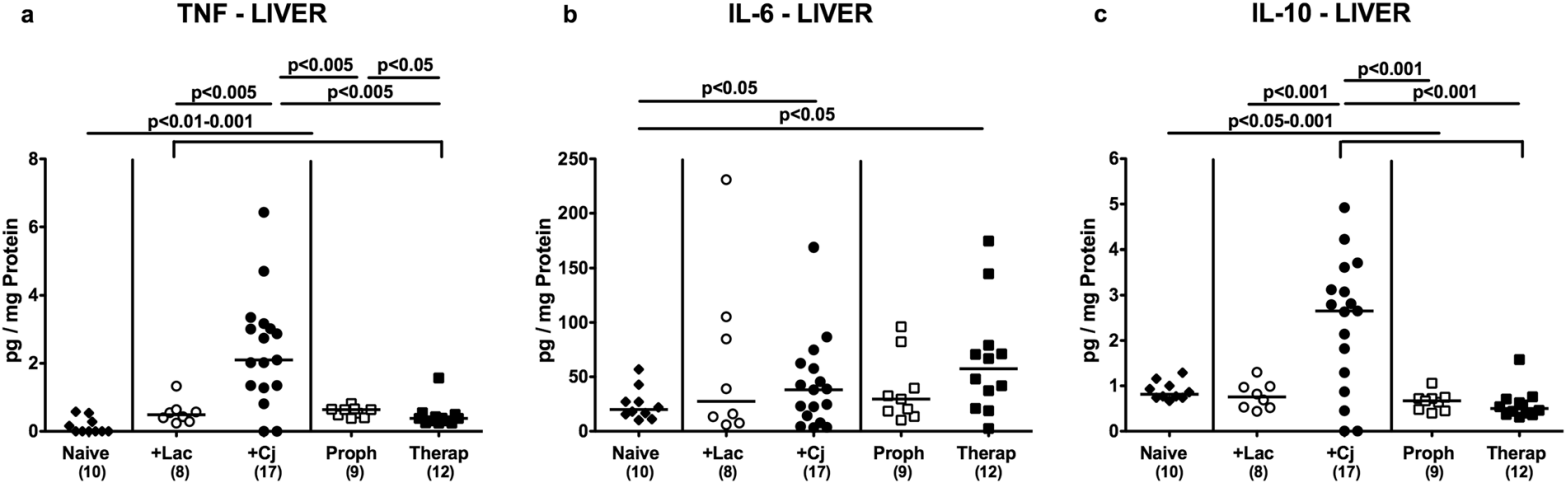
Secretion of pro- and anti-inflammatory mediators in livers of *C*. *jejuni* strain 81–176 and/or *L*. *johnsonii* reassociated secondary abiotic mice. Secondary abiotic mice were perorally infected with *C*. *jejuni* strain 81–176 (Cj) and associated with *L*. *johnsonii* (Lac) either 14 days before (prophylactic regimen, Proph; white squares) or 7 days thereafter (therapeutic regimen, Therap; black squares) and compared to mono-associated mice (+Lac, white circles; +Cj, black circles). (**a**) TNF, (**b**) IL-6 and (**c**) IL-10 concentrations were determined in *ex vivo* biopsies derived from livers at days 21 or 28 following initial *C*. *jejuni* or *L*. *johnsonii* infection, respectively. Naive (N) mice (black diamonds) served as uninfected controls. Medians (black bars), levels of significance (p-value) determined by one-way ANOVA test followed by Tukey post-correction test for multiple comparisons and numbers of analyzed animals (in parentheses) are indicated. Data were pooled from three independent experiments.

We next investigated whether potential immunomodulatory properties of *L*. *johnsonii* were restricted to the intestinal tract or could additionally even be observed in an extra-intestinal compartment such as the liver. TNF concentrations increased multifold in livers upon *C*. *jejuni* infection (p < 0.01–0.001; Fig. 6a). These increases, however, could be reduced by prophylactic as well as therapeutic co-administration of *L*. *johnsonii* (p < 0.005; Fig. 6a), that was most pronounced in the latter group. Moreover, increased hepatic IL-6 secretion could be determined upon *C*. *jejuni* infection (p < 0.05; Fig. 6b), except for with *L*. *johnsonii* prophylactically treated mice. Furthermore, IL-10 concentrations were elevated in livers following *C*. *jejuni* mono-, but not co-association with *L*. *johnsonii* (Fig. 6c). Hence, alleviation of *C*. *jejuni* induced pro-inflammatory immune responses by *L*. *johnsonii* was not restricted to the intestinal tract.

### Systemic cytokine responses upon reassociation of secondary abiotic mice with *C*. *jejuni* and/or *L*. *johnsonii*

**Figure 7:**
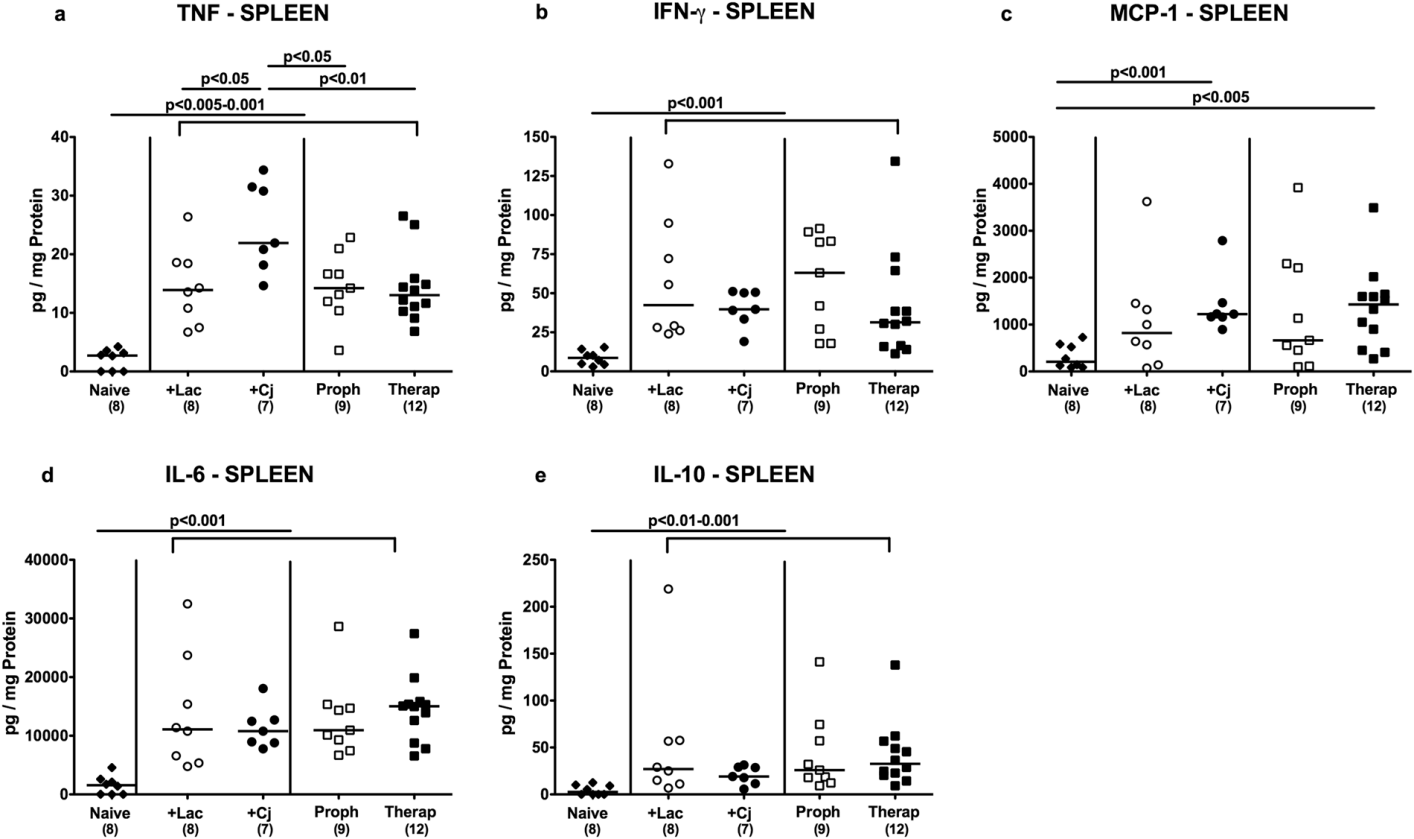
Splenic secretion of pro- and anti-inflammatory mediators of *C*. *jejuni* strain 81–176 and/or *L*. *johnsonii* reassociated secondary abiotic mice. Secondary abiotic mice were perorally infected with *C*. *jejuni* strain 81–176 (Cj) and associated with *L*. *johnsonii* (Lac) either 14 days before (prophylactic regimen, Proph; white squares) or 7 days thereafter (therapeutic regimen, Therap; black squares) and compared to mono-associated mice (+Lac, white circles; +Cj, black circles). (**a**) TNF, (**b**) INF-γ, (**c**) MCP-1, (**d**) IL-6, and (**e**) IL-10 concentrations were determined in *ex vivo* biopsies derived from spleens at days 21 or 28 following initial *C*. *jejuni* or *L*. *johnsonii* infection, respectively. Naive (N) mice (black diamonds) served as uninfected controls. Medians (black bars), levels of significance (p-value) determined by one-way ANOVA test followed by Tukey post-correction test for multiple comparisons and numbers of analyzed animals (in parentheses) are indicated. Data were pooled from three independent experiments.

**Figure 8:**
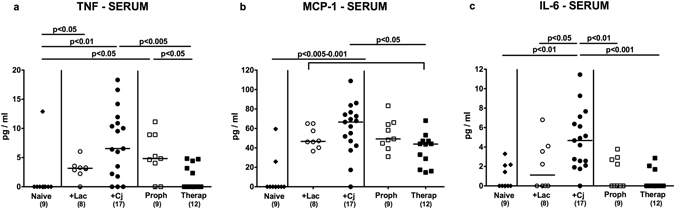
Pro-inflammatory cytokine secretion in serum of *C*. *jejuni* strain 81–176 and/or *L*. *johnsonii* reassociated secondary abiotic mice. Secondary abiotic mice were perorally infected with *C*. *jejuni* strain 81–176 (Cj) and associated with *L*. *johnsonii* (Lac) either 14 days before (prophylactic regimen, Proph; white squares) or 7 days thereafter (therapeutic regimen, Therap; black squares) and compared to mono-associated mice (+Lac, white circles; +Cj, black circles). (**a**) TNF, (**b**) MCP-1 and (**c**) IL-6 concentrations were determined in serum samples at days 21 or 28 following initial *C*. *jejuni* or *L*. *johnsonii* infection, respectively. Naive (N) mice (black diamonds) served as uninfected controls. Medians (black bars), levels of significance (p-value) determined by one-way ANOVA test followed by Tukey post-correction test for multiple comparisons and numbers of analyzed animals (in parentheses) are indicated. Data were pooled from three independent experiments.

Given the beneficial effects of *L*. *johnsonii* observed in extra-intestinal tissue sites of *C*. *jejuni* infected mice we next addressed whether respective immunomodulatory “probiotic” properties could also be observed systemically. We therefore measured cytokine secretion in spleen and serum samples derived from bacterial *in vivo* competition experiments. Following bacterial reassociation, splenic TNF, IFN-γ, IL-6 as well as IL-10 concentrations increased multifold (p < 0.01–0.001; Fig. 7a,b,d,e). Notably, *C*. *jejuni* induced TNF levels could be lowered by prophylactic and therapeutic coassociation with *L*. *johnsonii* (p < 0.05 and 0.01, respectively; Fig. 7a). In addition, neither *L*. *johnsonii* mono-association nor prophylactic co-association of *C*. *jejuni* infected mice was associated with increased splenic MCP-1 levels (n.s. vs naive mice; Fig. 7c). Furthermore, increased TNF serum concentrations could be measured upon *C*. *jejuni* infection alone and following prophylactic, but not therapeutic *L*. *johnsonii* co-association of *C*. *jejuni* infected mice (Fig. 8a). Whereas bacterial reassociation was accompanied by increased MCP-1 concentrations in serum (p < 0.005–0.001; Fig. 8b), *C*. *jejuni* induced increases in serum MCP-1 levels could be lowered by therapeutic *L*. *johnsonii* treatment (p < 0.05; Fig. 8b). In line with this, prophylactic as well as therapeutic *L*. *johnsonii* co-association reduced *C*. *jejuni* induced increases in serum IL-6 levels to basal levels (p < 0.01 and p < 0.001, respectively; Fig. 8c). Notably, neither viable *L*. *johnsonii* nor *C*. *jejuni* could be isolated from extra-intestinal and systemic compartments including liver, spleen and blood at necropsy (not shown). Hence, *L*. *johnsonii* administration ameliorated not only intestinal and extra-intestinal, but also systemic *C*. *jejuni* induced pro-inflammatory cytokine responses.

## Discussion

Probiotics are effective in the treatment of gastrointestinal diseases as supported by a multitude of scientific investigations including clinical studies^31^. Proposed mechanisms of beneficial probiotic action include modification of the intestinal microbiota^32^, enhancement of colonization resistance^26^, support of intestinal barrier functions^33^ as well as modulation of innate and adaptive immunity^34^. In case of campylobacteriosis, probiotics (in particular lactobacilli) have been shown to co-aggregate with *C*. *jejuni* thus reducing the adhesion to and invasion of the intestinal epithelial cell layers and to suppress motility which is essential for pathogenicity^24, 35^. The modes of actions of the corresponding probiotic formulations have been examined for decades. For instance, administration of heat killed bacteria of the closely related *L*. *acidophilus* reduced translocation of *C*. *jejuni* in infected isolator-raised germfree mice^36^.

Given that *L*. *johnsonii* and its relative *L*. *acidophilus* have been shown to produce several antimicrobial molecules including bacteriocins and peroxide^37^, we addressed in *in vivo* bacterial competition experiments shown here, whether pathogenic loads could be lowered upon *L*. *johnsonii* application before and/or after *C*. *jejuni* infection of secondary abiotic mice. Both probiotic and pathogenic strains could stably colonize the gastrointestinal tract at high loads upon peroral challenge. Unfortunately, neither therapeutic nor prophylactic *L*. *johnsonii* application could decrease intestinal *C*. *jejuni* burdens in a biologically relevant fashion. In a previous *in vivo* study applying isolator-raised germfree mice that had been reconstituted with a complex human microbiota, a complete eradication of *C*. *jejuni* from the small and large intestines could be observed upon pretreatment of human microbiota-associated mice with a probiotic mix of *L*. *acidophilus*, *L*. *reuteri* and *Bifidobacterium infantis* ^26^. One needs to take into consideration, however, that the observed contrasting pathogen-eradicative outcomes could most likely not only be explained by the quantitative and qualitative differences in the used probiotic strains, but also by substantially different immunological features of the applied mouse models. Due to the lacking contact to any bacterial ligands and subsequent absence of immunological differentiation and stimulation, isolator-raised germfree mice exhibit only poorly-developed intestinal lymphatic tissues^38, 39^, and need thus to be regarded as highly immunocompromized and inappropriate for dissecting commensal intestinal-pathogen-host interactions. The secondary abiotic mice used in our study, however, were born, reared and maintained under conventional conditions and exhibited a fully developed innate and adaptive immune system. Notably, recolonization of secondary abiotic mice with a single probiotic strains might not be sufficient to reconstitute the complex physiological prerequisites (i.e. colonization resistance) for effective competition with *C*. *jejuni* for nutrients and niches. Thus, rather a combination of several beneficial microbes instead of one single probiotic strain would be a more promising strategy to protect from or combat established enteropathogenic infections.

Whereas in our study re-colonized mice were not clinically/macroscopically compromized, *C*. *jejuni* infection induced colonic apoptosis and inflammation. Notably, *L*. *johnsonii* colonization alone did not induce apoptotic, but promoted proliferative/regenerative colonic epithelial responses that might counteract potential inflammation-attributed cell damage. Most importantly, the *C*. *jejuni* induced disease state could be ameliorated by treatment with *L*. *johnsonii*, which impacted apoptosis and adaptive immune responses upon *C*. *jejuni* infection. Again, *L*. *johnsonii* alone did not lead to an influx of innate or adaptive immune cells into the colonic mucosa and lamina propria. Furthermore, increases in intestinal pro-inflammatory mediators (including IL-6, NO and TNF) induced by *C*. *jejuni* infection could be alleviated by peroral *L*. *johnsonii* co-administration. Notably, respective beneficial immunomodulatory effects were not restricted to the large intestinal tract, but could also be observed in the ileum. As compared to uninfected control mice, *L*. *johnsonii* application alone further resulted in enhanced IL-10 secretion in intestinal as well as systemic compartments as shown from *ex vivo* biopsies taken from MLN and spleen, respectively. Our *in vivo* data are further supported by results from *in vitro* studies. Tsuda and colleagues reported, for instance, that stimulation of intestinal lymphocytes (derived from MLN of isolator-raised germfree mice) with a mix of three defined and inactivated *L*. *johnsonii* strains induced IL-10 production and regulated excessive antigen-specific cytokine responses via antigen presenting cells^40^. In line with this, stimulation of peritoneal macrophages with *L*. *johnsonii* also resulted in enhanced IL-10 secretion^41^. In weanling as well as malnourished aged mice, *L*. *johnsonii* challenge further enhanced intestinal IgA secretion, thus constituting recovery and/or modulation of accelerated immune responses within the intestinal tract^42, 43^.

In our study *L*. *johnsonii* administration did not only ameliorate intestinal, but also extra-intestinal such as hepatic, and most strikingly, systemic *C*. *jejuni* induced pro-inflammatory cytokine responses. In support, a study applying a murine leukemia model revealed that peroral restoration of commensal *Lactobacillus* spp. (that were reduced upon disease-mediated dysbiosis) resulted in decreased levels of pro-inflammatory cytokines including IL-6 and MCP-1 in muscular as well as serum samples^44^. Given that the efficacy of *L*. *johnsonii* as therapeutic and/or preventive measure directed against enteric diseases including infections is supported by solid data from experimental *in vitro* and *in vivo* investigations as well as clinical studies^37^, it has been included in probiotic products distributed worldwide (Nestlé LC1).

**In conclusion**, even though neither susceptibility to *C*. *jejuni* infection nor pathogenic burdens upon intestinal establishment were influenced by *L*. *johnsonii* co-administration, the anti-inflammatory properties of this single probiotic species that became overt in intestinal, extra-intestinal and, remarkably, systemic compartments of *C*. *jejuni* infected mice qualifies *L*. *johnsonii* at least as adjunct immunomodulatory application. It is rather a well-orchestrated interplay of mucosal immunity and the intestinal intraluminal milieu determined by the concert of the complex microbiota plus beneficial probiotic strains that is required to successfully combat and/or prevent from enteropathogenic infections. Future studies are needed, however, to better understand the underlying mechanisms.

## Material and Methods

### Ethics statement

All animal experiments were conducted according to the European Guidelines for animal welfare (2010/63/EU) with approval of the commission for animal experiments headed by the “Landesamt für Gesundheit und Soziales” (LaGeSo, Berlin, registration number G0184/12). Animal welfare was monitored twice daily by assessment of clinical conditions including weight loss.

### Generation of secondary abiotic mice

Female C57BL/6j mice were reared and housed within the same specific pathogen free (SPF) unit in the Forschungseinrichtungen für Experimentelle Medizin (FEM, Charité - University Medicine Berlin). Secondary abiotic mice were generated by quintuple antibiotic treatment for eight weeks starting 10 weeks of age as described before^45^. Mice were kept in cages including filter tops within an experimental semi-barrier (accessible only with lab coat, overshoes, caps and sterile gloves) under standard conditions (22–24 °C room temperature, 55 ± 15% humidity, 12 h light/12 dark cycle). Mice were handled under aseptic conditions and had unlimited access to autoclaved tap water and chow (food pellets; Sniff, Soest, Germany). Murine samples were taken at comparable time points in the morning.

### Bacterial *in vivo* competition experiments

Three days before bacterial reassociation experiments the antibiotic cocktail was replaced by sterilized tap water. Mice were then perorally challenged by gavage with either *C*. *jejuni* strain 81–176 or with a commensal *L*. *johnsonii* strain that had initially been isolated from the intestinal tract of a healthy female 3 months-old C57BL/6j wildtype mouse (respective bacterial loads of 10^8^ CFU) as described previously^12, 45^. Briefly, single colonies of lactic acid bacteria were isolated from murine feces samples by cultivation of serial dilutions on MRS selective agar (Oxoid, Wesel, Germany). The capacity of the isolates to inhibit growth of *C*. *jejuni* was examined by co-cultivation assays with *C*. *jejuni* strain 81–176 on Columbia agar supplemented with 5% sheep blood (Oxoid) and in thioglycolate liquid media (Bachelor thesis Ulrike Escher, Beuth Hochschule, Berlin, Germany), according to standard microbial stabbing techniques for isolation of lactobacilli which are inhibitory for pathogens^46^. The isolate that mediated the most extensive growth inhibition of *C*. *jejuni* strain 81–176 in both assays was further investigated. The corresponding pleomorphic Gram-positive rods did neither produce catalase nor oxidase and were identified as *L*. *johnsonii* by molecular analysis of the complete 16S rRNA gene sequence.

For bacterial *in vivo* competition experiments, *C*. *jejuni* infected mice were either challenged with *L*. *johnsonii* 7 days thereafter (therapeutic regimen) or 14 days before (prophylactic regimen). Naive sex and age-matched animals served as uninfected (negative) controls. Mice were continuously kept in a sterile environment (autoclaved food and drinking water or sterile antibiotic cocktail *ad libitum*) and were handled under strict aseptic conditions in order to prevent from contaminations.

### Clinical Score

To survey clinical signs of inflammation, a standardized cumulative clinical score (maximum 12 points), addressing the occurrence of blood in feces (0: no blood; 2: microscopic detection of blood by the Guajac method using Haemoccult, Beckman Coulter/PCD, Krefeld, Germany; 4: overt blood visible), diarrhea (0: formed feces; 2: pasty feces; 4: liquid feces), and the clinical aspect (0: normal; 2: ruffled fur, less locomotion; 4: isolation, severely compromized locomotion, pre-final aspect) was applied daily as described earlier^47^.

### Sampling procedures

Mice were sacrificed 21 or 28 days following initial *C*. *jejuni* or *L*. *johnsonii* reassociation, respectively, by isofluran treatment (Abbott, Greifswald, Germany). Cardiac blood and tissue samples from the colon, ileum, MLN, liver and spleen were removed under sterile conditions. Intestinal samples were collected in parallel for microbiological and immunological analyses. Immunohistological changes were assessed in colonic *ex vivo* biopsies that had been immediately fixed in 5% formalin and embedded in paraffin. Sections (5 μm) were stained with distinct antibodies for *in situ* immunohistochemistry as described earlier^14, 47^.

### Quantitative analysis of pathogenic and probiotic bacterial colonization and translocation

*C*. *jejuni* strain 81–176 and *L*. *johnsonii* were quantitated in feces over time post reassociation or upon necropsy in luminal samples taken from the colon, in homogenates of whole tissue *ex vivo* biopsies derived from spleen and liver (approximately 1 cm^3^) and in cardiac blood (approximately 1 mL). Whereas cardiac blood was analyzed by direct plating, feces, luminal colon contents and organ homogenates were dissolved in sterile phosphate buffered saline (PBS; Gibco life technologies, Paisley, UK) and serial dilutions cultured on Karmali and Columbia agar supplemented with 5% sheep blood (Oxoid) for two days at 37 °C under microaerobic conditions using CampyGen gas packs (Oxoid) for *C*. *jejuni* detection as described earlier^12^. *L*. *johnsonii* loads were determined on Columbia agar supplemented with 5% sheep blood, Columbia-CNA agar supplemented with colistin and nalidixic acid (both Oxoid), and MRS agar (Oxoid) in parallel and incubated under microaerobic (in jars using CampGen gas packs; Oxoid) and obligate anaerobic (in jars using Anaerogen gas packs; Oxoid) conditions for at least two days. Bacterial species were identified according to their typical morphological appearances and sequencing of the 16S rRNA genes. The detection limit of viable bacteria was ≈100 CFU per g.

### Immunohistochemistry

*In situ* immunohistochemical analysis of colonic paraffin sections was performed as described earlier^14, 47^. Primary antibodies against cleaved caspase-3 (Asp175, Cell Signaling, Boston, MA, USA, 1:200), Ki67 (TEC3, Dako, Glostrup, Denmark, 1:100), CD3 (#N1580, Dako, Denmark, dilution 1:10), FOXP3 (FJK-16s, eBioscience, San Diego, CA, USA, 1:100), B220 (eBioscience, 1:200) and F4/80 (# 14–4801, clone BM8, eBioscience, 1:50) were used. For each animal the average number of positively stained cells within at least six high power fields (HPF, 400x magnification) were determined microscopically by an independent blinded investigator.

### Cytokine measurements in intestinal and extra-intestinal compartments

Colonic and ileal *ex vivo* biopsies were cut longitudinally and washed in PBS. MLN, spleen or strips of approximately 1 cm^2^ of colon, ileum and liver tissues were placed in 24-flat-bottom well culture plates (Nunc, Wiesbaden, Germany) containing 500 μL serum-free RPMI 1640 medium (Gibco) supplemented with penicillin (100 U/mL) and streptomycin (100 µg/mL; PAA Laboratories, Cölbe, Germany). After 18 h at 37 °C, culture supernatants were tested for TNF, IFN-γ, MCP-1, IL-6, IL-12p70 and IL-10 by the Mouse Inflammation Cytometric Bead Assay (CBA; BD Biosciences, Heidelberg, Germany) on a BD FACSCanto II flow cytometer (BD Biosciences). NO was determined by the Griess reaction as described earlier^45^.

### Statistical analysis

Medians, means and significance levels using appropriate tests as indicated (Mann Whitney U test and one-way ANOVA with Tukey’s post hoc test for multiple comparisons) were determined using GraphPad Prism Software v6 (La Jolla, CA, USA). Two-sided probability (p) values ≤ 0.05 were considered significant. Experiments were repeated twice.

## Electronic supplementary material

Supplemental Figure S1
