# Supplementary material for: Lactobacillus johnsonii ameliorates intestinal, extra-intestinal and systemic pro-inflammatory immune responses following murine Campylobacter jejuni infection
Source: Sci Rep. 2017 May 18;7:2138. doi: 10.1038/s41598-017-02436-2 (PMC5437126; doi:10.1038/s41598-017-02436-2)
Supplement: Supplementary file 1 — Supplemental Figure S1 [file 41598_2017_2436_MOESM1_ESM.pdf]

Lactobacillus johnsonii ameliorates intestinal, extra-intestinal and systemic pro-inflammatory immune responses following murine *Campylobacter jejuni* infection

Stefan Bereswill, Ira Ekmekciu, Ulrike Escher, Ulrike Fiebiger, Kerstin Stingl, and Markus M. Heimesaat

Apoptotic Cells (Casp3+)

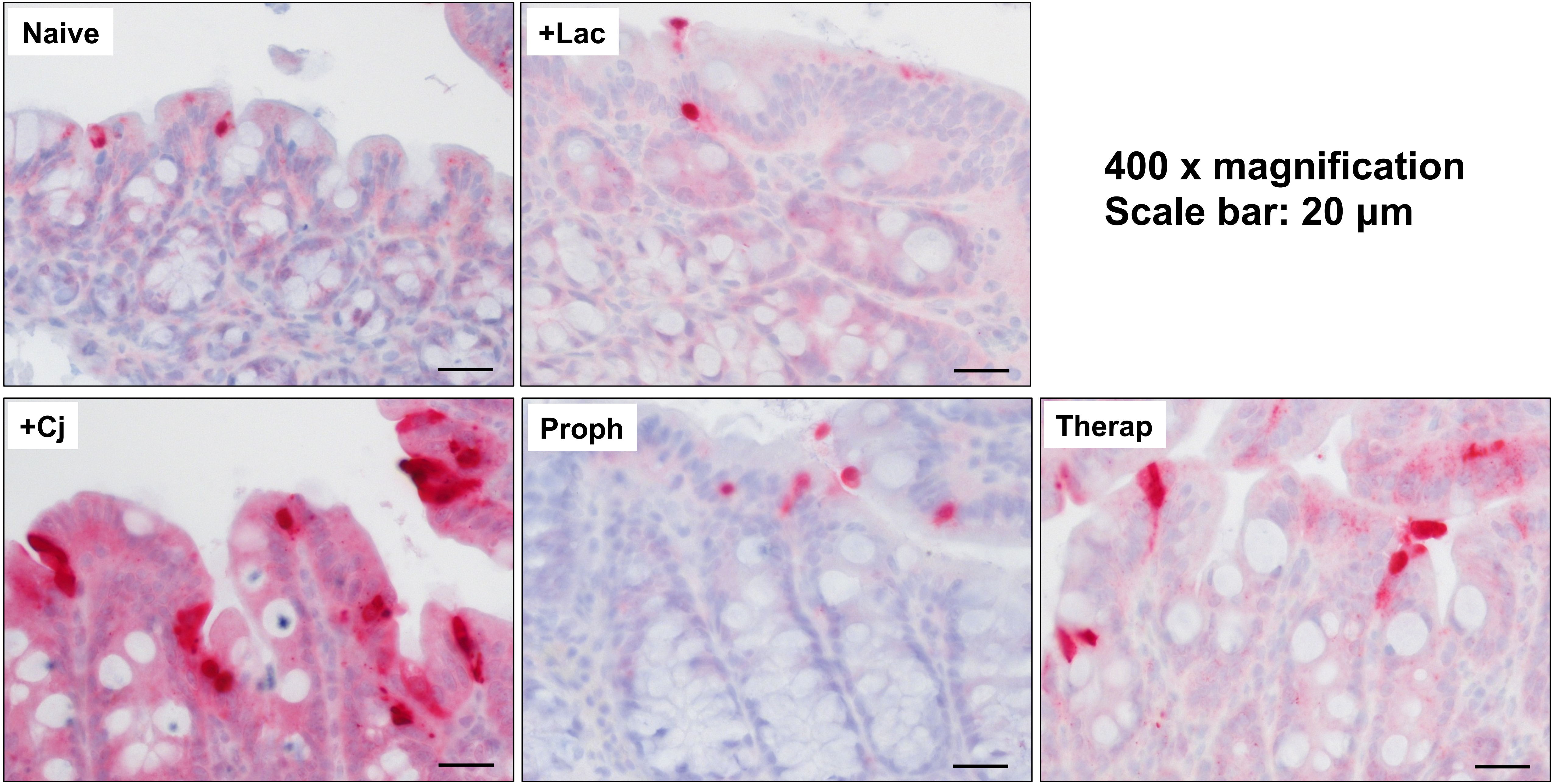

400 x magnification
Scale bar: 20 µm

Proliferating Cells (Ki67+)

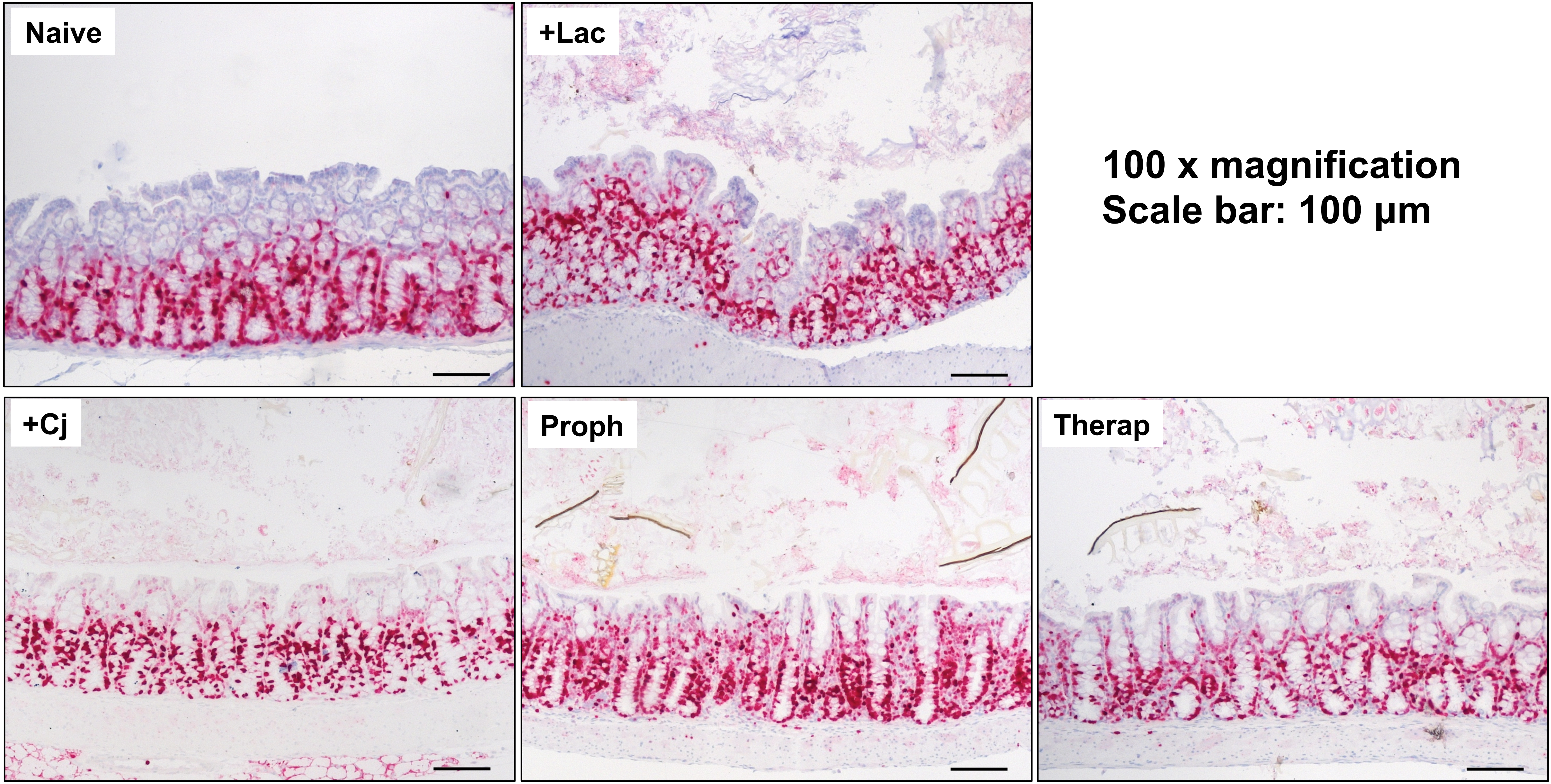

100 x magnification
Scale bar: 100 µm

T Lymphocytes (CD3+)

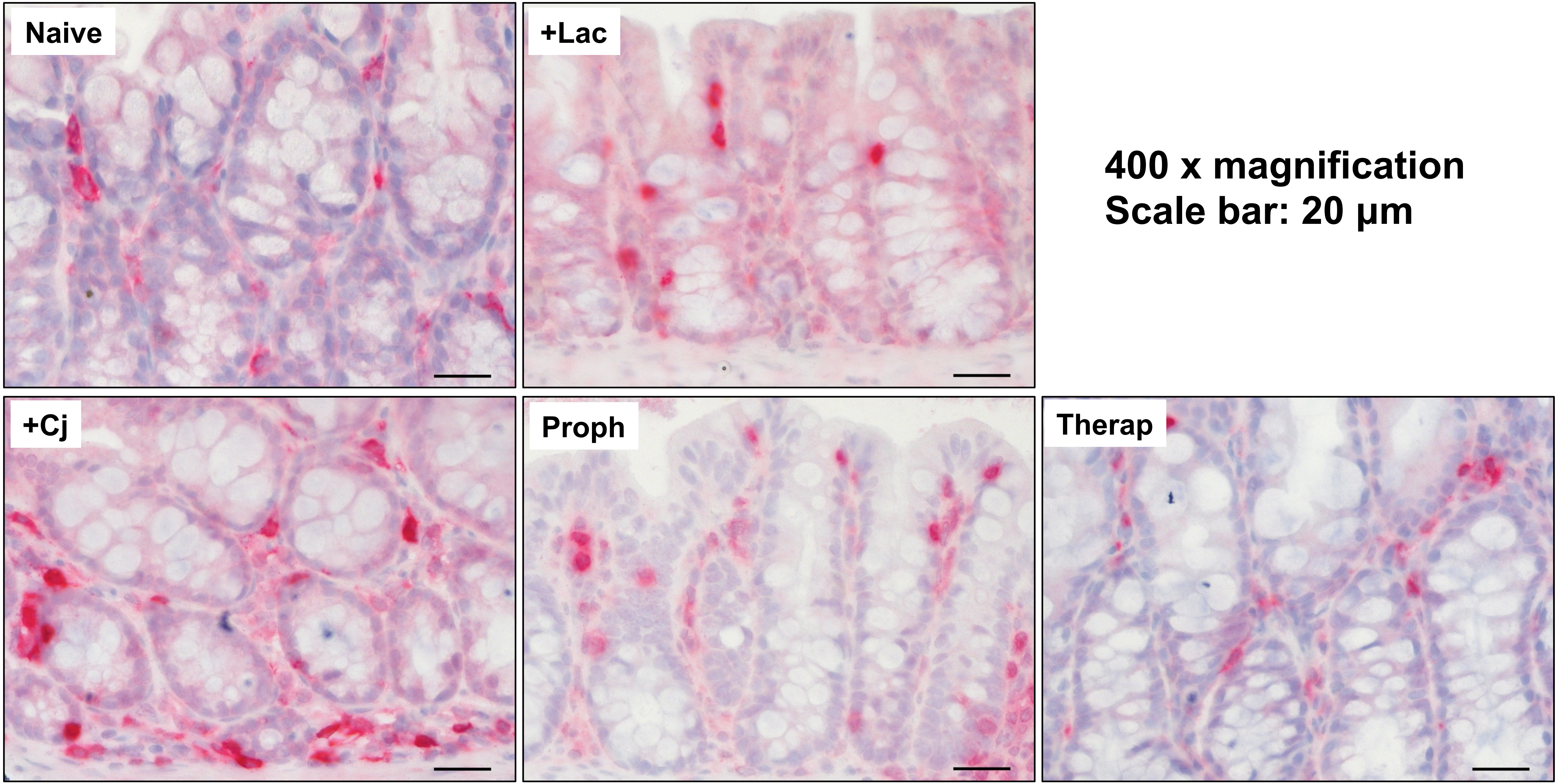

400 x magnification
Scale bar: 20 µm

Regulatory T Cells (Treg, FOXP3+)

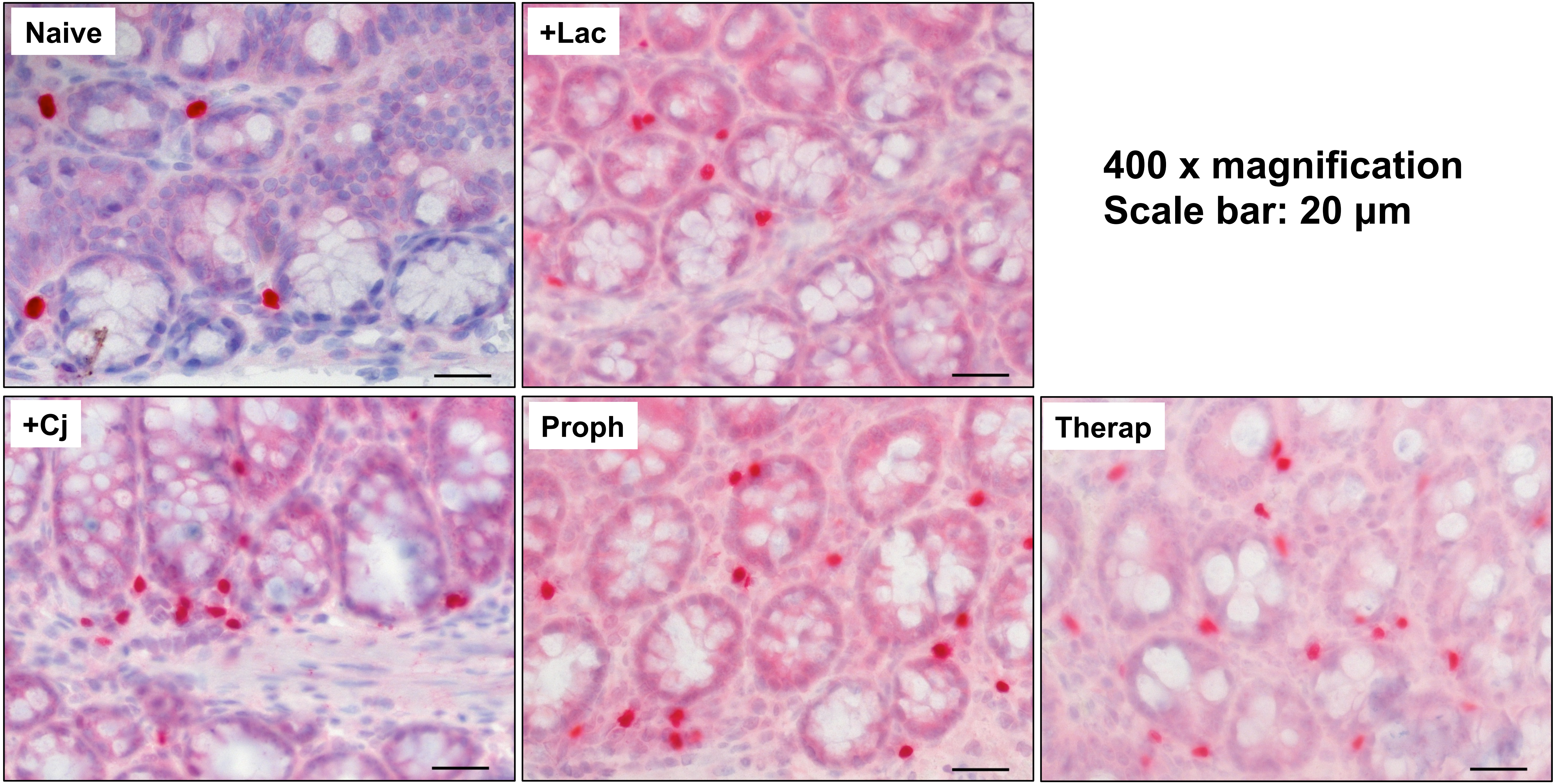

400 x magnification
Scale bar: 20 µm

B Lymphocytes (B220+)

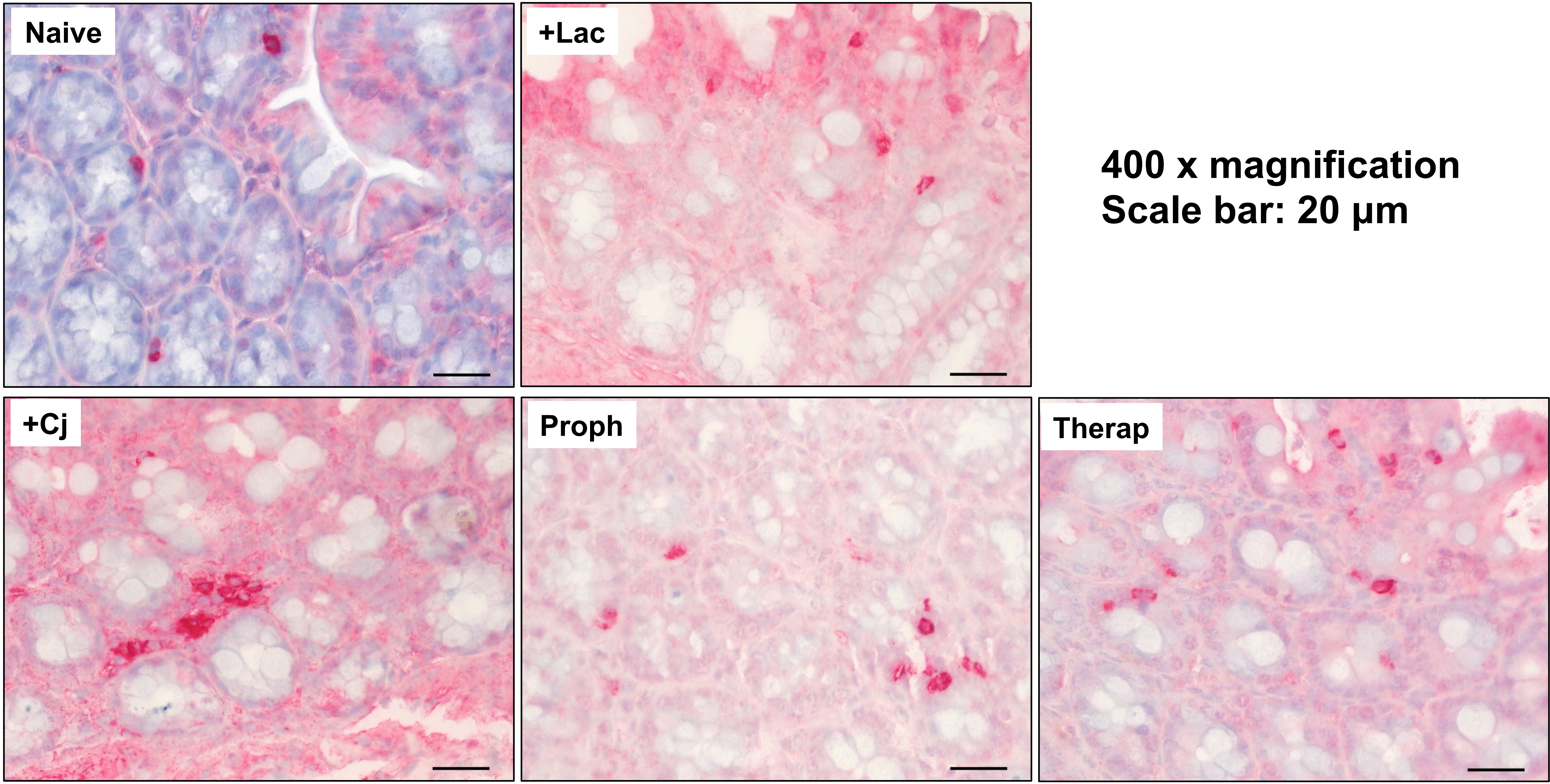

400 x magnification
Scale bar: 20 µm

Macrophages / Monocytes (F4/80+)

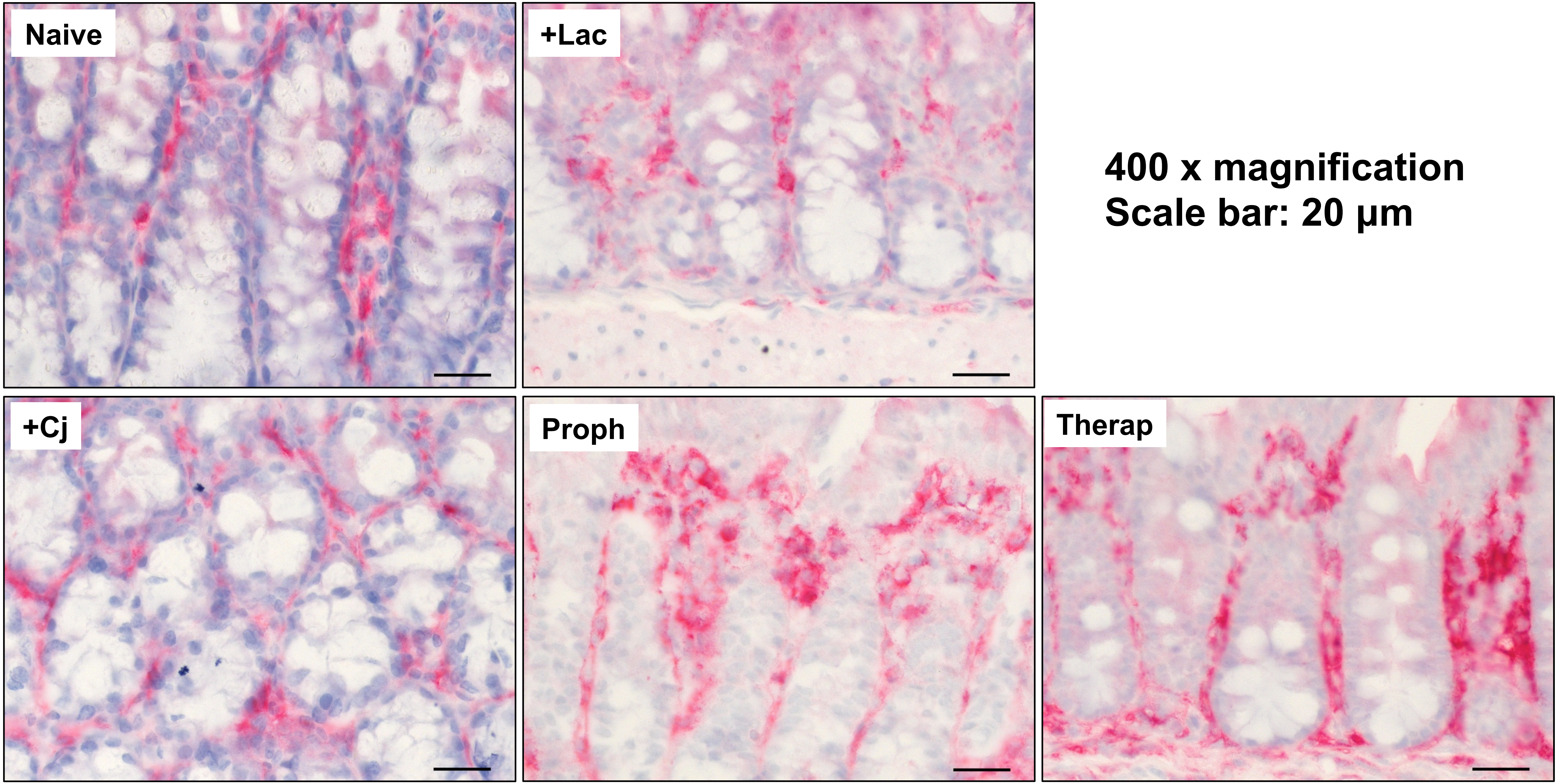

400 x magnification
Scale bar: 20 µm
